# Birth-Rate and Sex Education

**Published:** 1920-04-10

**Authors:** 


### Birth-Rate and Sex Education.

The National Birth-rate Commission is to continue its inquiry. Its terms of reference are very important, and comprise a wide and difficult range of subjects. They include consideration of the development and education of young citizens for worthy parenthood, under the following heads:—

1. The various methods of educating boys and girls in sex hygiene before they leave the home and school, and the extent to which graded instruction in sex matters can be usefully given by parents, school teachers, ministers of religion, physicians, and others.
2. Those influences and conditions which favour or retard the bodily and mental development of the adolescent citizen, in so far as these are concerned with the attainment of worthy parenthood.
3. The extent to which worthy ideals of citizenship and parenthood can and should be inculcated by education in its widest sense.

Other matters included in the terms of reference are the influence of various industrial occupations on the birth-rate; the housing problem; schemes for the "endowment of motherhood," and widows' pensions; problems of migration within the Empire; new discoveries in dietetics; the relation of religious belief to the birth-rate; the 1921 census; and the co-ordination of inquiries in Great Britain and the Dominions with those in the United States, France, and other countries.

